# Denial of Risk Behavior Does Not Exclude Asymptomatic Anorectal Sexually Transmitted Infection in HIV-Infected Men

**DOI:** 10.1371/journal.pone.0008504

**Published:** 2009-12-30

**Authors:** Edward R. Cachay, Amy Sitapati, Joseph Caperna, Kellie Freeborn, Joseph T. Lonergan, Edward Jocson, William C. Mathews, for the Owen Clinic Study Group

**Affiliations:** School of Medicine, University of California San Diego, San Diego, California, United States of America; Karolinska Institutet, Sweden

## Abstract

**Background:**

The Centers for Disease Control recommend screening for asymptomatic sexually transmitted infection (STI) among HIV-infected men when there is self-report of unprotected anal-receptive exposure**.** The study goals were: (1) to estimate the validity and usefulness for screening policies of self-reported unprotected anal-receptive exposure as a risk indicator for asymptomatic anorectal infection with Neisseria gonorrhoeae (GC) and/or Chlamydia trachomatis (CT). (2) to estimate the number of infections that would be missed if anal diagnostic assays were not performed among patients who denied unprotected anorectal exposure in the preceding month.

**Methods and Findings:**

Retrospective analysis in HIV primary care and high resolution anoscopy (HRA) clinics. HIV-infected adult men were screened for self-reported exposure during the previous month at all primary care and HRA appointments. Four sub-cohorts were defined based on microbiology methodology (GC culture and CT direct fluorescent antibody vs. GC/CT nucleic acid amplification test) and clinical setting (primary care vs. HRA). Screening question operating characteristics were estimated using contingency table methods and then pooled across subcohorts. Among 803 patients, the prevalence of anorectal GC/CT varied from 3.5–20.1% in the 4 sub-cohorts. The sensitivity of the screening question for self-reported exposure to predict anorectal STI was higher in the primary care than in the HRA clinic, 86–100% vs. 12–35%, respectively. The negative predictive value of the screening question to predict asymptomatic anorectal STI was ≥90% in all sub-cohorts. In sensitivity analyses, the probability of being an unidentified case among those denying exposure increased from 0.4–8.1% in the primary care setting, and from 0.9–18.8% in the HRA setting as the prevalence varied from 1–20%.

**Conclusion:**

As STI prevalence increases, denial of unprotected anal-receptive exposure leads to an increasingly unacceptable proportion of unidentified asymptomatic anorectal STI if used as a criterion not to obtain microbiologic assays.

## Introduction

The Centers for Disease Control (CDC) recommendation for anorectal screening for asymptomatic sexually transmitted infection (STI) among HIV-infected men who have sex with men (MSM) is “to test for rectal infection with Neisseria gonorrhoeae (GC) and Chlamydia trachomatis (CT) in men who acknowledge anal-receptive intercourse during the preceding year” [1]. Similarly, the Infectious Diseases Society of America (IDSA) recommends rectal testing for GC/CT infection “on the basis of report of receptive anal intercourse” [2]. According to these recommendations, screening for asymptomatic anorectal STI depends on the patient's self-report of unprotected anal-receptive exposure. In the absence of anorectal symptoms related to an STI, patients may not perceive themselves to be at risk and may therefore not request screening. Furthermore, from the medical provider's perspective, microbiological ascertainment of asymptomatic anorectal STI is a relatively time consuming procedure in busy HIV primary care settings [3].

The objectives of this study were to estimate:(1) the prevalence of asymptomatic anorectal STI in an HIV-infected male population under care;(2) the sensitivity, specificity, positive and negative predictive values of self-reported unprotected anorectal exposure in the preceding month for anorectal infection with GC and/or CT; and (3) the number of infections that would be missed if anal diagnostic assays were not performed among patients who denied unprotected anorectal exposure in the preceding month.

## Materials and Methods

The study was a retrospective analysis conducted in a HIV primary care clinic and its co-located high resolution anoscopy (HRA) clinic at the University of California, San Diego (UCSD). All study participants were adult HIV-positive men. This study was conducted according to the principles expressed in the Declaration of Helsinki. The study was approved by the Institutional Review Board of the UCSD Human Research Protection Program, project# 071931. All patients provided written informed consent for the collection of samples and subsequent analysis.

As part of routine care, a brief self-report screening instrument for unprotected anorectal exposure during the previous month was implemented at all primary care and HRA clinic visits. The self-administered assessment of recent sexual risk behaviors is available at: http://health.ucsd.edu/owenclinic/PatientSurveyForms.html. The initial evaluation of the screening tool was previously published [4]. The questions used to screen for unprotected anorectal exposure were: (a) in the primary care clinic, “During the past month, did your partner put his penis in your anus without using a condom even once?” (screening considered positive for “yes” response) and (b) in the HRA clinic, “During the last month, with how many people have you had anal-receptive sex without using a condom?”(screening considered positive for one or more partner) The selection of these items was based on patient focus group findings and pilot testing for acceptability and comprehension. The surveys were explained by the medical assistant at the time vital signs were recorded and then self- administered on paper by the patient. The completed forms were given to the clinician and it was the responsibility of the clinician to obtain microbiologic samples during the primary care visit or HRA procedure based on agreed upon screening criteria (see below).

In the primary care clinic, patients were seen at least three times per year. Selective screening for anal STI was recommended based on the self-reported numbers of sex partners in the preceding month (30-day period). In the primary care cohort between September 2003- June 2008, screening was recommended if the patient reported at least 1 sex partner during the preceding month. Beginning July 2008, screening was recommended if the patient reported at least 3 sex partners during the preceding month. In the HRA clinic, collection of rectal swabs for STI is a routine part of the procedure for all patients.

Before July 2008, microbiologic investigation for anorectal CT and GC included direct fluorescent antibody (DFA) assay, (PathoDx Diagnostic Products Corporation, Los Angeles, Calif.) and cultures using modified Thayer-Martin media, respectively [5]. In July 2008, UCSD completed the validation process for the use of nucleic amplification tests (NAAT) of anorectal samples for diagnosis of CT and GC infections according to Clinical Laboratory Improvement Amendments (CLIA) regulations [6]. In both settings, we switched to NAAT assays for diagnosis of anorectal CT and GC infections. The NAAT used was the Becton Dickinson ProbeTec ET (Sparks, MD) assay, which has a sensitivity of 76.5–77.8% and a specificity above 99% for diagnosis of rectal GC and CT infections among MSM, respectively [7], [8]. All patients who tested positive for GC or CT in any microbiology assay were treated according to the standard of care.

Four sub-cohorts were defined based on a) methods of microbiologic diagnostic assays: GC culture and CT DFA vs. GC and CT NAAT; and b) clinical setting: primary care vs. HRA clinic. Because the four sub-cohorts differed according to inclusion criterion, clinical setting, screening-question expression, and assay methodology, we analyzed each separately and then pooled across subcohorts after examining heterogeneity of effect.

Screening question operating characteristics were estimated as sensitivity (SE), specificity (SP), positive predictive value (PPV), and negative predictive value (NPV) for asymptomatic anorectal STI. The pooled SE and SP of self-reported unprotected anorectal exposure were estimated using a random-effects model implemented in STATA version 11 (StataCorp, College Station, Texas, USA).Heterogeneity among sub-cohorts was assessed using the I-squared statistic [9] and heterogeneity chi-squared test. Finally, to estimate the probability of unidentified anorectal STI among patients who denied unprotected exposure in the preceding month if anal diagnostic assays were not performed (1–NPV), we conducted sensitivity analyses based on the pooled screening question SE and SP from our data, varying the prevalence of asymptomatic anorectal STI from 1–20%. We also modeled, under varying prevalence, the number of undiagnosed asymptomatic anorectal STI cases in a hypothetical cohort of 1000 patients under the assumption that those not disclosing exposure would not be screened with a microbiologic assay, while those acknowledging exposure would all be screened with an assay with 100 percent sensitivity and specificity to detect anorectal gonorrhea and Chlamydia trachomatis infection. Hypothesis tests were 2-sided with an alpha error of 0.05.

## Results

Between September 2003 and May 2009, 803 male patients (40.2% of all males under care) were screened: 38.2% were non-white, 82% reported MSM as their main HIV risk factor, and median CD4 and log_10_-transformed HIV viral load were 441 (Interquartile range [IQR]: 298–643) and 1.70 (IQR: 1.68–2.03), respectively. The study participants were divided into four sub-cohorts: a) Primary care clinic screened using GC culture and CT DFA; b) Primary care clinic screened using NAAT; c) HRA clinic screened using GC culture and CT DFA; and d) HRA clinic screened using NAAT. Patients enrolled in the four sub-cohorts completed a total of 1,155 screening visits (Table 1).

**Table 1:** Sub-cohorts for screening of asymptomatic anorectal STI according to clinical setting and microbiology assay methodologies.

Cohort	Testing method	Study period	N° Men n = 803	N° visits n = 1,155	Screening question
Primary care clinic	Culture for GC, DFA CT	8/2/05–7/10/06	55	58	“During the past month, did your partner put his penis in your anus without using a condom even once?”
Primary care clinic	NAAT GC/CT	11/1/08–3/31/09	34	34	“During the past month, did your partner put his penis in your anus without using a condom even once?”
HRA clinic	Culture for GC, DFA CT	9/9/03–6/30/05	425	597	“During the last month, with how many people have you had anal-receptive sex without using a condom?” (0 vs.≥1)
HRA clinic	NAAT GC/CT	8/4/08–11/10/09	408	466	“During the last month, with how many people have you had anal-receptive sex without using a condom?” (0 vs.≥1)

STI = Sexually transmitted infection. HRA = High Resolution Anoscopy. GC = Neisseria gonorrhoeae. CT = Chlamydia trachomatis. DFA = Direct fluorescent antibody assay.

NAAT = Nucleic acid amplification test.

In the two primary care sub-cohorts, clinician compliance with screening recommendations was sub-optimal. In the primary care sub-cohort using culture and DFA, 271 patients completed the screening questionnaire but only 58 (21%) underwent microbiologic assessment. The 213 (79%) with missing microbiology assays appeared to be at lower STI risk compared to those with complete data based on reported prevalence of at least one episode of unprotected anorectal exposure during the preceding month (39 vs. 71%, p<0.0001, respectively). For the primary care NAAT sub-cohort, 11 of 45 (24%) lacked microbiologic assay results. They also appeared to be at lower STI risk than those with complete data based on self-reported unprotected anorectal exposure during the preceding month (36 vs.71%, p = 0.07, respectively).

### Prevalence of Asymptomatic Anorectal STI

The prevalence of asymptomatic anorectal STI varied according to screening eligibility criteria. For the two HRA sub-cohorts (universal screening) and the primary care sub-cohort requiring *at least one sex partner* during the previous month, the prevalence of positive assays for anorectal GC and/or CT infection varied from 3.4–4.3% and was similar regardless of assay methodology (Table 2). However, for the primary care sub-cohort enrolled using selective screening with a requirement of *at least 3 sex partners* during the preceding month, the prevalence was 21% (95% confidence interval[CI]:9–38) using NAAT. Of note, the prevalence of asymptomatic anorectal STI varied according to number of reported partners during the prior month in the HRA NAAT sub-cohort:3, 8 and 15% for 0, 1 or ≥2 self reported partners, respectively [χ^2^ (2d.f.) 9.298, p  = 0.010]. We found no association between number of reported partners and anorectal STI prevalence in the other three sub-cohorts, conditional upon sub-cohort eligibility criteria.

**Table 2:** Prevalence of asymptomatic anorectal STI and operating characteristics of screening questions to predict asymptomatic anorectal STI by sub-cohort.

Cohort & setting	Percentage Disclosing Exposure	Prevalence Anal STI	Screening Question Sensitivity	Screening Question Specificity	Screening Question NPV	Screening Question PPV	TP (n)	FN (n)	FP (n)	TN (n)
Primary care Clinic using GC culture/ CT DFA	70.7 (57.2 –81.9)	3.4 (0.4–11.9)	100 (15.8–100)	30.4 (18.8–44.1)	100 (80.5–100)	4.9 (0.60–16.5)	2	0	39	17
Primary care clinic using NAAT	70.6 (52.5–84.9)	21 (8.7–37.9)	85.7 (42.1–99.6)	33.3 (16.5–54)	90 (55.5–99.7)	25 (9.7–46.7)	6	1	18	9
HRA clinic using GC culture/CT DFA	17.9 (14.9 –21.2)	4.2 (2.7–6.1)	12 (2.6–31.2)	81.8 (78.4–84.9)	95.5 (93.3–97.2)	2.8 (0.6–8)	3	22	104	468
HRA clinic using NAAT	14.8 (11.7–18.4)	4.3 (2.6–6.6)	35 (15.4–59.2)	86.1 (82.5–89.2)	96.7 (94.5–98.2)	10.1 (4.2–19.8)	7	13	62	384

Results expressed in percentage with parenthesis denoting 95 percentage confidence interval for each value.

Note: PPV = Positive predictive value. NPV = Negative predictive value. STI = sexually transmitted infection. HRA = High resolution anoscopy. GC = Neisseria gonorrhoeae. CT = Chlamydia trachomatis. DFA = Direct fluorescent antibody assay.

NAAT = Nucleic acid amplification test.

TP = true positive. FN = false negative. FP = false positive. TN = true negative. n = number.

### Operating Characteristics of the Screening Questions

The SE of the screening question for self-reported unprotected anorectal exposure to predict anorectal STI was consistently higher in the primary care setting than in the HRA clinic, 86–100% vs. 12–35%, respectively (Table 2). In contrast, the SP of the screening question to predict a STI was lower in the primary care setting (30–33%) than in the HRA setting (82–86%). The PPV was unsurprisingly low in all studied sub-cohorts (2.8–25%) given that the majority of patients reporting unprotected anorectal exposure do not acquire anorectal STI. However, the NPV of the screening question to predict an asymptomatic anorectal STI was 90% or above among all sub-cohorts (Table 2).

### Pooling of Screening Question Operating Characteristics

**Figure 1:**
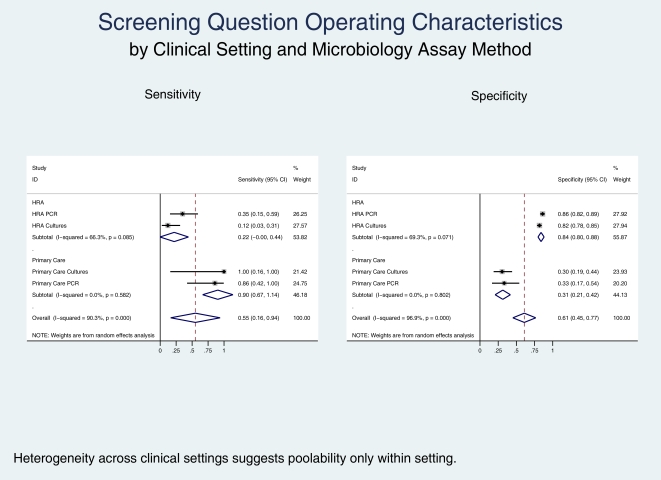
Sensitivities and specificities of screening question to predict asymptomatic anorectal STI according to clinical setting (‘primary care clinic vs. high resolution anoscopy clinic’) and microbiology assay method (‘GC culture and CT direct fluorescent antibody vs. GC/CT nucleic acid amplification test’).

There was substantial heterogeneity of effect among the four sub-cohorts (; I-squared inconsistency 90.3%, p<0.001). Therefore, based on examination of operating characteristics of individual sub-cohorts, we pooled separately the two primary care and the two HRA sub-cohorts (Figure 1). For the primary care sub-cohorts, the pooled SE and SP were 90 and 31%, respectively. For the HRA sub-cohorts, the pooled SE and SP were 22 and 84%, respectively.

### Sensitivity Analysis

**Figure 2:**
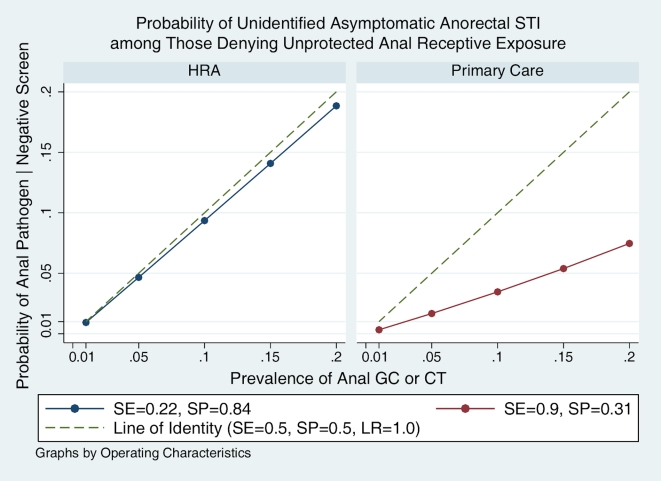
Sensitivity analysis of the probability of unidentified asymptomatic anorectal STI among those denying unprotected anal receptive exposure. X-axis displays prevalence of asymptomatic STI (C. trachomatis and/or N. gonorrhoeae infection). Y-axis shows the probability defined as one minus negative predictive value of the screening question.

**Figure 3:**
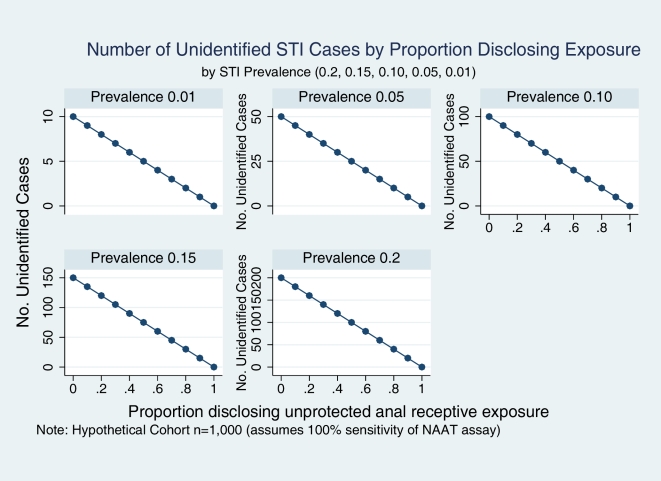
Model to estimate the number of unidentified cases based on a hypothetical cohort of 1000 patients, stratified by prevalence of asymptomatic STI (0.01, 0.05, 0.10, 0.20). Model assumes that those disclosing unprotected anal receptive exposure would all be screened with a NAAT assay having 100% sensitivity and specificity.

These pooled estimates of SE and SP were applied in sensitivity analyses to estimate the probability of unidentified asymptomatic anorectal STI among those denying exposure and therefore not undergoing microbiologic screening. This probability can be interpreted as one minus the negative predictive value of the screening question. We observed that as the prevalence of anorectal STI varies from 1–20% in settings similar to the primary care sub-cohorts in this study (pooled SE 90%, SP 31%), the probability of being an unidentified case among those denying exposure increases from 0.4–8.1%. In contrast, in settings similar to the HRA sub-cohorts (SE 22%, SP 84%), as the prevalence varies from 1–20%, the probability of being an unidentified case increases from 0.9–18.8%, respectively (Figure 2). To estimate the number of unidentified cases, we performed an analysis based on a hypothetical cohort of 1000 patients assuming that those disclosing exposure would all be screened with a NAAT assay having 100% sensitivity and specificity. The analysis was stratified by prevalence of asymptomatic STI (0.01, 0.05, 0.10, 0.20). In our scenario, if all patients who experienced unprotected anorectal exposure disclosed exposure, all would be screened and all cases identified. This would result in sensitivity for the screening question of 100%. At the other extreme, if all who experienced unprotected anorectal intercourse denied exposure, none would be screened and no cases would be identified. The sensitivity of the screening question would then be 0% (Figure 3). In our study, we found that the proportion disclosing exposure was considerably lower in the HRA clinic (14.8–17.9%) than in the primary care clinic (70.6–70.7%).

## Discussion

The present study to assess the utility of self-disclosed unprotected anorectal exposure to predict asymptomatic anorectal STI found that:(1) the operating characteristics of the studied screening questions were highly dependent upon screening setting (primary care vs. HRA);(2) in all sub-cohorts, the prevalence of self-reported unprotected anal-receptive intercourse varied considerably by setting (primary care higher than HRA) but was homogeneous within setting;(3) the number of self-disclosed sexual partners in the preceding month may be a useful screening selection criterion to identify patients at high risk for asymptomatic anorectal STI;(4) despite agreement among primary care clinicians to obtain anorectal microbiologic assays for patients identified through behavioral screening to be at risk, the compliance rate was poor with evidence that those with highest exposure risk were selectively screened in comparison with those at lower exposure risk; and (5) modeling the unidentified STI cases as the post behavioral screening probability of STI (1–NPV) and as the absolute number of unidentified cases highlights the importance of background prevalence of anorectal STI and the proportion of patients acknowledging anorectal risk behavior as key determinants of the impact of a selective screening policy that limits microbiologic testing to those acknowledging exposures.

### Screening Setting Effect

The screening questions for unprotected anal- receptive exposure performed more poorly in the HRA setting than in the primary care setting. This could be attributed to different factors other than setting itself. First, the questions were framed differently in the two settings. In the primary care setting, the question was part of a comprehensive sexual risk behavior instrument and asked if there was even a single episode of unprotected anal-receptive exposure during the prior month. In the HRA setting, the question was imbedded in a brief questionnaire dealing with factors related to the scheduled high resolution anoscopy and asked about the number of partners with whom the patient had unprotected anal-receptive exposure during the prior month. Second, in the primary care setting, the patients completed the questionnaire as part of a visit that was otherwise unrelated to an anogenital examination whereas in the HRA setting the patients were asked to complete the questionnaire prior to an anticipated anorectal examination.

### Differential Prevalence of Disclosed Risk Behaviors

We noted a striking difference in self-reported prevalence of unprotected anal-receptive exposure between the two primary care sub-cohorts (approximately 70%) and the two HRA sub-cohorts (15–18%). While it is possible that these differences in reported prevalence reflect differences in actual risk behavior [10]–[12], we believe it likely that other factors are operative. In favor of the differences being real, one could hypothesize that those attending the HRA clinics, knowing that they had abnormal anal cytology and were being referred to the HRA clinic, may have reduced their risk behaviors during the month preceding the examination. The prevalence of STI (3.5–4.3%) was similar in both HRA sub-cohorts and the primary care sub-cohort requiring for inclusion only one acknowledged sex partner during the prior month. This would suggest that either actual behaviors over the risk periods were similar in these 3 sub-cohorts or that asymptomatic anorectal STI was prevalent for a longer period of time in the HRA sub-cohorts. An alternative explanation for the varying prevalence of reported risk behaviors in the two clinical settings is differential functioning of *social desirability bias* due to differences in question framing, screening context and perceived normative expectations [13]. We believe it is unlikely that patients would over-report sexual risk behaviors in either clinical context (tending to decrease the specificity of the screening question) but would rather tend to under-report risk behaviors in both settings (tending to decrease sensitivity of the screening question). Empirical research has identified methods to improve accuracy of responses to intrusive or sensitive questions. A key finding of that research is that self-administration of survey items is preferred to interviewer-administration [14]. But it is not simply the physical presence of another person during survey administration that increases the probability of biased responding. “What seems to matter is the threat that someone with whom the respondent will continue to interact (an interviewer or bystander) will learn something embarrassing about the respondent” [14]. During STI screening, if self-reported risk behaviors are included in the screening algorithm, it is essential to determine the actual risk-behavior history. Relying only on clinicians, however, to assess risk behaviors and then selectively screen with microbiology assays is unlikely to result in a high-fidelity screening program.

### Number of Partners as Risk Identifier

The high prevalence of asymptomatic anal STI in the primary care sub-cohort employing NAAT could be due to the investigator decision to limit eligibility to those men acknowledging at least three sex partners in the prior month. We looked for consistency in this association *post hoc* analytically in the three other sub-cohorts and found a statistically significant association between partner number and STI prevalence in the HRA NAAT sub-cohort but not in the other two sub-cohorts. While the association is biologically plausible and consistent with other research [15]–[17], there was no consistency among the sub-cohorts. A higher observed prevalence in the primary care NAAT cohort could have been due to a period effect of higher STI prevalence in the community such that the same number of exposures would be more likely to result in infection independent of the number of partners involved.

### Poor Clinician Compliance with Screening Recommendations

A recent survey that examined data from HIV-infected MSM receiving care at six cities in the United States from 2004 through 2006 found that only 5% of patients under care had been screened for anorectal STI [3]. Our data from the primary care sub-cohorts illustrates the challenge of integrating routine anorectal-STI screening into HIV care settings. 24 and 79% of patients with behavioral risk information lacked microbiologic data in the primary care NAAT and primary care culture/DFA sub-cohorts, respectively. We found that implementing routine behavioral screening during the check-in process, supervised by a medical assistant, greatly improved the documentation of risk behaviors in comparison to a strategy that relied on clinician initiative to document (data not shown). However, obtaining anal specimens for microbiologic assay can only be performed by a qualified clinician.

### Modeling Undiagnosed Cases

The proportion of all STI cases not identified in a screening question evaluation is estimated as the false negative proportion (1–SE). The absolute number of unidentified cases depends on the false negative proportion of the screening question and on the prevalence of asymptomatic STI. In contrast, the post-behavioral screen probability of STI (1–NPV) is dependent on both the disease prevalence and the screening question operating characteristics (SE, SP) [18]. Based on our data, we estimated that the proportion of all cases that would be missed if microbiologic screening were limited to patients acknowledging risk behavior during the prior month varied from 11% in the primary care setting to 78% in the HRA setting. The impact in terms of absolute numbers of missed cases is directly related to disease prevalence as illustrated in Figure 3, and, under a worst case scenario in which all patients denied exposure, could vary in a hypothetical cohort of 1,000 from 10 missed cases (STI prevalence 1%) to 200 cases (STI prevalence 20%). The range of asymptomatic anal STI prevalence found in this study is similar to those reported in other recent studies of MSM, including those that are HIV infected [6], [19]–[24]. What proportion or absolute number of missed cases should trigger universal screening regardless of acknowledged risk is best addressed by cost-effectiveness analyses that include both risks for further transmission as well as morbidity in the index patients. Another question not addressed by our data is the determination of an appropriate screening interval and whether such an interval can be reliably based on reported risk behaviors.

A number of additional limitations of our analysis should be considered. First, we assumed that the microbiologic assays performed as a perfect gold standard [25]. But published sensitivities of the assays utilized were not perfect. Notwithstanding, we have no reason to believe that the pattern of false negative microbiologic assays would be differentially associated with reported risk behaviors. Assuming that the screening questions and microbiologic assays were conditionally independent, a bias correction can be applied to adjust for the imperfect gold standard assays [26]. The maximum absolute difference between unadjusted and adjusted screening question sensitivity and specificity across all four sub-cohorts was 0.08. With regard to specificity of the NAAT assay, although quite high (>99%), our clinicians did not routinely follow up positive NAAT results with culture prior to treatment. Second, in both the primary care and HRA settings, the screening question recall period was 1 month. To the extent that asymptomatic anorectal STI could be present for longer than 1 month, the sensitivity of the screening question would be further compromised by risk behaviors resulting in transmission prior to the 1 month window. The longest documented duration of asymptomatic anorectal gonococcal infection, to our knowledge, was 165 days [27]. The CDC recommends a one-year risk behavior recall period as part of the recommended screening criteria [1]. However, in most HIV primary care settings patients are routinely seen every 4 months as recommended by IDSA guidelines [2]. Research in sexual risk behaviors has shown that extending the question recall period beyond one month may increase the likelihood of recall bias and make the data less reliable especially in high risk populations such as the one that we studied [28]–[29]. Third, we did not include syphilis or sexually transmitted enteric pathogens in our definition of asymptomatic anorectal STI.

### Conclusions

Under the conditions used in this study, denial of unprotected anal-receptive exposure would lead, in our opinion, to an unacceptable number and proportion of unidentified asymptomatic anorectal STI if used as a criterion not to obtain microbiologic assays. The sensitivity and specificity of behavioral risk screening questions can vary markedly by clinical setting and by question framing. Improvement in screening question performance should be based on findings of empirical research regarding survey ascertainment of sensitive or stigmatized risk behaviors. Structuring primary care encounters to facilitate routine collection of anorectal microbiologic assays, even for patients with self-disclosed risk behaviors, remains an ongoing challenge.
